# After the game is before the game!

**DOI:** 10.3205/zma001329

**Published:** 2020-04-15

**Authors:** Christoph Nikendei

**Affiliations:** 1 Universitätsklinik für Allgemeine Innere Medizin und Psychosomatik, Sektion Psychotraumatologie, Heidelberg, Germany; 2 Patrick-Henry-Village Heidelberg, Geflüchtetenambulanz des Zentrums für Psychosoziale Medizin der Universitätsklinik Heidelberg, Heidelberg, Germany

## Editorial

Dear Readers, 

“The ball is round, and a game lasts 90 minutes.” (Sepp Herberger). And we know what to do. We have no choice. We throw everything forward. Every woman, every man. Even if we are acting defensively and time is running out. We go all in – we almost completely blow our cover. We know what to do: nearly anything – really anything. “The round thing has to go into the square thing.” (Sepp Herberger). It's that simple. With this clarity, concentration and stormy composure, thousands of scientists, health and nursing professionals, doctors and members of many other healthcare professions are currently working – no fighting the battle against a much smaller opponent – a tiny one: the new coronavirus 2019-nCoV [1], which cannot even be stopped by the tight netting of a mouth and nose protector, let alone a football net.

Faced with the current situation, it is simply unthinkable to write a medical text without making a reference to this pandemic. Too much suffering, too many exhausted helpers, too many overwhelming experiences. 375,498 confirmed cases and 16,362 deaths in 195 countries and territories have been recorded according to the latest WHO statistics (as of 25.03.2020; [https://experience.arcgis.com/experience/685d0ace521648f8a5beeeee1b9125cd]. In Wuhan, people are cautiously hesitant to leave their shelters again, while in other places they are waiting in fear – with reports from Spain and Italy breathing down their necks – for the first offshoots of the great wave of sick and respirator-dependent people. We are all preparing ourselves – bracing ourselves for the unknown. We have one of the best healthcare systems. We are well educated. 

Without wanting to overtax the power of association: This issue, too, reflects part of a mosaic of many thousands of individual conceptual and scientific achievements which, taken together, will help us to overcome – and I am sure we will overcome – this current crisis. The scope of this issue ranges from interactive learning methods for improved knowledge acquisition in microscopic anatomy [2], the contribution of international mobility to medical professionalization [3] and the evaluation of interprofessional attitudes [4], to the illumination of the long-term evaluation of the medical didactic qualification within the framework of the Master of Medical Education (MME; [5]), the optimization of the evaluation of clinical-practical trials [6], the strengthening of outpatient care through the curricular implementation of learning content [7], programs to strengthen rural care [8], and train-the-trainer programs for the qualification of continuing education officers in general practice [9]. It is precisely these didacticians, teachers, scientists and authors who are making their contribution so that we do not find ourselves powerless in the face of a crisis.

Yes, they are. As if that were not enough, I am putting you through even more today. Because at the moment, in view of the many deaths caused by the corona crisis, all the stress on the helpers and the social insecurity in society, it is hard to name and believe: the corona crisis will be far eclipsed by another crisis: the climate crisis. The Lancet Countdown on health and climate change [10] has made this very clear: the health of society in future and the generations to come depends entirely on how we deal with climate change today. According to the latest study by the World Bank, 140 million climate refugees [11] will be added to the 70 million refugees currently in the world by 2050 – combined with enormous despair and suffering [12]. By the end of the current century, depending on the type of cereal, we will have seen harvests decline by up to 19% [13] and will be faced with the question of how to feed the 7.7 billion people currently living on earth. Extreme meteorological events, such as storms, floods, high water, heavy rainfall and heat waves, will cause accidents with traumatic injuries or fatalities [14] and up to 70% of the people affected will suffer trauma [15]. 8-12% of the average mortality of the total population will increase during heat waves [16], with an increase in the average earth temperature by a further 1.5°C, 1000 additional heart attacks will occur in Germany each year [17]. Phenomena, like “eco-anxiety” will continue to spread in a manner requiring treatment [18].

We are unable to see the virus. But we can all feel the rising temperature, we can all see the effects of rising sea levels, we can all hear the reports of droughts. Yet these issues are underrepresented in our medical curricula [19]. Here too, as in the Corona crisis, we must learn to listen to and trust the scientific knowledge we have been so wise in fostering and nurturing ourselves [20], even if “the truth, through its incredibility, threatens to elude detection”. (Heraclitus). This is a central task of us scientists, doctors, medical didactics and lecturers. “We are staying you! Stay home for us!” is the current motto. We have the chance of learning from the corona crisis in a way that will help us address our biggest threat: the climate crisis “by design” instead of “by disaster” as is currently the case, i.e. reacting in a controlled and targeted manner in a similarly solidarity as we are successfully mustering today instead of letting ourselves be overrun by the disaster which is inevitably coming. So as in football: we need to attack. Immediately. With all our strength. “After the game is before the game!” (Sepp Herberger). Otherwise, we are in danger of resuming the thread of a global pan-suicide [21] after the corona crisis has been overcome.

Stay healthy, everyone, 

Yours, Christoph Nikendei

## Competing interests

The author declares that he has no competing interests.
